# Comparison of Functional and Hemodynamic Parameters Between Methamphetamine-Associated and Idiopathic Pulmonary Arterial Hypertension: Systematic Review and Meta-Analysis

**DOI:** 10.3390/healthcare14142089

**Published:** 2026-07-13

**Authors:** Fani Papaioannou, Thomas Karagiannis, Afroditi Boutou, Athina Georgopoulou, Stergios Kaprinis, Georgia Pitsiou

**Affiliations:** 1 Respiratory Failure Department, G Papanikolaou Hospital, Aristotle University of Thessaloniki, 57010 Thessaloniki, Greece; afboutou@auth.gr (A.B.); gr.athina@yahoo.gr (A.G.); gpitsiou@auth.gr (G.P.); 2 Clinical Research and Evidence-Based Medicine Unit, Second Medical Department, Aristotle University of Thessaloniki, 54642 Thessaloniki, Greece; tkaragian@auth.gr; 3 Second Department of Psychiatry, Aristotle University of Thessaloniki, 56429 Thessaloniki, Greece; skaprinis@auth.gr

**Keywords:** functional status, hemodynamics, idiopathic pulmonary arterial hypertension, meta-analysis, methamphetamine-associated pulmonary arterial hypertension, systematic review

## Abstract

**Background:** We conducted a systematic review and meta-analysis to compare functional and hemodynamic parameters between patients with Meth-APAH and idiopathic PAH (IPAH). **Methods:** MEDLINE, Scopus, and gray literature were searched up to September 2025. Eligible studies were observational and compared functional and hemodynamic parameters between patients with Meth-APAH and IPAH. All participants had undergone diagnostic right heart catheterization (RHC) and were classified by methamphetamine use history and/or toxicological screening. Primary outcomes included World Health Organization Functional Class III or IV (WHO-FC III/IV), six-minute walk distance (6-MWD), and mean pulmonary arterial pressure (mPAP). Secondary outcomes were cardiac index (CI), right atrial pressure (RAP), pulmonary vascular resistance (PVR), stroke volume index (SVI), pulmonary artery wedge pressure (PAWP), and heart rate (HR). Random-effects models were applied for pooled estimates. **Results:** Five studies comprising 1991 participants were included. Patients with Meth-APAH demonstrated a significantly higher risk of being classified as WHO-FC III/IV (RR 1.17, 95%CI 1.05–1.30, *p* = 0.01), as well as higher RAP (MD 1.59, 95%CI 0.07–3.11, *p* = 0.04) and reduced SVI (MD −3.57, 95%CI −6.74–−0.4, *p* = 0.04), but differences were modest. No significant differences were found for 6-MWD, mPAP, CI, PVR, PAWP, or HR. **Conclusions:** Meth-APAH could be associated with worse functional and hemodynamic profile compared to IPAH, but available studies are few with high heterogeneity, so that clear differences in several functional and hemodynamic parameters could not be established between these disease entities. These results highlight the need for further research in this direction.

## 1. Introduction

Methamphetamine is a potent synthetic psychostimulant that belongs to the amphetamine class of psychoactive substances [1]. Due to its structural similarity to monoamines, methamphetamine can substitute for dopamine, norepinephrine, and serotonin at their respective transporters (DAT, NET, and SERT) in both the central and autonomic nervous systems, reversing their function while also inhibiting vesicular monoamine transporter 2 (VMAT-2) [2].

Globally, it is estimated that approximately 35 million people use amphetamine-type stimulants, with methamphetamine being the predominant substance, making it the third most commonly abused drug [3]. At low to moderate doses, methamphetamine induces euphoria, whereas frequent and high doses are associated with the onset of psychotic episodes and cardiovascular complications [1,2].

Methamphetamine has now been established as a definitive risk factor for the development of pulmonary arterial hypertension (PAH) [4]. Methamphetamine-associated PAH (Meth-APAH) is classified under drug- and toxin-associated PAH, a subgroup that accounts for approximately 9.5–10.5% of all PAH cases [5,6].

The mechanisms underlying Meth-APAH are multifactorial. Methamphetamine accumulates in pulmonary tissue, altering serotonergic signaling, promoting oxidative and endoplasmic reticulum stress, and inducing endothelial apoptosis and proliferation of pulmonary artery smooth muscle cells (PASMCs) [3,7,8]. Recent studies have implicated genetic susceptibility, particularly polymorphisms in the carboxylesterase 1 (CES1) encoding gene, and estrogen–serotonin interactions in modulating disease risk [9,10,11]. Emerging molecular data also highlight inflammatory pathways involving lipocalin-2 and the NLRP3 (NOD-, LRR-, and pyrin domain-containing protein 3) inflammasome as potential mediators of pulmonary vascular remodeling [12].

The diagnostic approach for Meth-APAH follows the general diagnostic algorithm for PAH. As there are no specific clinical guidelines for Meth-APAH, management generally follows the treatment algorithm proposed for idiopathic, heritable, drug-induced, and connective tissue disease-associated PAH, yet treatment access and adherence remain major challenges in this population [4,13,14]. Prognostic data regarding Meth-APAH remain limited and often conflicting, with most authors agreeing that certain hemodynamic parameters tend to be less favorable in Meth-APAH compared to idiopathic PAH (IPAH) [13,14,15,16,17].

Given the growing burden of methamphetamine use and the limited evidence in this field, we conducted a systematic review and meta-analysis, for the first time in the literature, in order to compare functional and hemodynamic parameters between patients with Meth-APAH and those with IPAH.

## 2. Methods

The conduct and reporting of this review adhere to the guidelines for Preferred Reporting Items for Systematic reviews and Meta-Analyses (PRISMA) [18]. The study protocol was registered in the international prospective register of systematic reviews (PROSPERO) under the registration number CRD420251157591.

### 2.1. Search Strategy

A systematic literature search was conducted in the MEDLINE (via PubMed) and Scopus databases, up to 1 September 2025. Efforts were made to include all available studies by thoroughly screening the reference lists of retrieved articles and by manually searching for gray literature, including conference proceedings. The search strategy incorporated both controlled vocabulary terms (Medical Subject Headings, MeSH) and free-text keywords, including “methamphetamine”, “methamphetamine-associated”, “methamphetamine-associated pulmonary arterial hypertension”, “pulmonary arterial hypertension”, “PAH”, “idiopathic pulmonary arterial hypertension”, “idiopathic PAH”, and “idiopathic pulmonary hypertension”. The full search strategy is provided in the Supplementary Materials.

### 2.2. Study Selection

Search results were imported into Mendeley Reference Manager (Version 2.125.0) for organization and removal of duplicate records. The remaining studies were independently screened at the title and abstract level by two reviewers to exclude irrelevant publications. Full-text screening was subsequently conducted to identify studies eligible for inclusion in the synthesis. Eligible studies were observational in design and compared patients with Meth-APAH and IPAH with respect to functional and hemodynamic parameters. Inclusion required that the diagnosis of PAH had been confirmed by right heart catheterization (RHC), and subgroup classification was based on documented methamphetamine use through patient history and/or toxicological testing. Articles published in languages other than English were excluded. Abstracts of unpublished studies were included when deemed relevant and when they provided sufficient data for analysis. Any discrepancies between reviewers were resolved through discussion and consensus.

### 2.3. Data Extraction

Data extraction from the included studies was performed independently by two reviewers and recorded in a standardized electronic Excel spreadsheet. Predefined data fields were used and included general study characteristics such as publication year, study design, sample size, participant age and sex, as well as data on primary outcomes, including the proportion of patients classified as World Health Organization Functional Class III or IV (WHO-FC III/IV), six-minute walk distance (6-MWD), and mean pulmonary arterial pressure (mPAP), and secondary outcomes, including cardiac index (CI), right atrial pressure (RAP), pulmonary vascular resistance (PVR), stroke volume index (SVI), pulmonary artery wedge pressure (PAWP), and heart rate (HR).

### 2.4. Quality Assessment

The methodological quality of the included studies was assessed using the JBI Critical Appraisal Checklist for Analytical Cross-Sectional Studies, as the meta-analysis was based on baseline measurements rather than longitudinal outcomes. Studies were evaluated based on the clarity of inclusion criteria, the description of participants and study settings, the presence of confounding factors and how they were addressed, the criteria used to assess the condition, the validity and reliability of exposure measurement, the measurement of outcomes, and the appropriateness of the statistical analysis [19]. Response options included “yes” and “no,” indicating high and low quality, respectively, as well as “unclear” and “not applicable.” All assessments were performed independently by two reviewers, each blinded to the other’s decisions.

### 2.5. Statistical Analysis

Data were synthesized using the inverse variance random effects model, due to the anticipated heterogeneity between studies. Continuous outcomes were described using the mean difference (MD) or standardized mean difference (SMD) as appropriate. Between-study variance (τ^2^) was estimated using the restricted maximum likelihood (REML) method. For dichotomous outcomes, the risk ratio (RR) was calculated, and τ^2^ was estimated using the Paule–Mandel method. Ninety-five percent confidence intervals (95% CIs) were calculated using the Hartung–Knapp adjustment. Statistical significance was defined as a *p*-value < 0.05. Heterogeneity across studies was assessed using the I^2^ statistic and Cochran’s Q test. The threshold *p* < 0.10 was set as statistically significant for the presence of heterogeneity, and I^2^ values greater than 50% were interpreted as indicating substantial heterogeneity. To evaluate the robustness of the findings, sensitivity analyses were performed by excluding matched studies, conference abstracts, and studies with substantial imbalance in sample size between the Meth-APAH and IPAH groups. All statistical analyses were conducted using R Studio (Version 2025.05.1+513) and specifically the “meta” package. Assessment of publication bias was not performed due to the small number of included studies.

## 3. Results

### 3.1. Search Results and Study Characteristics

**Figure 1:**
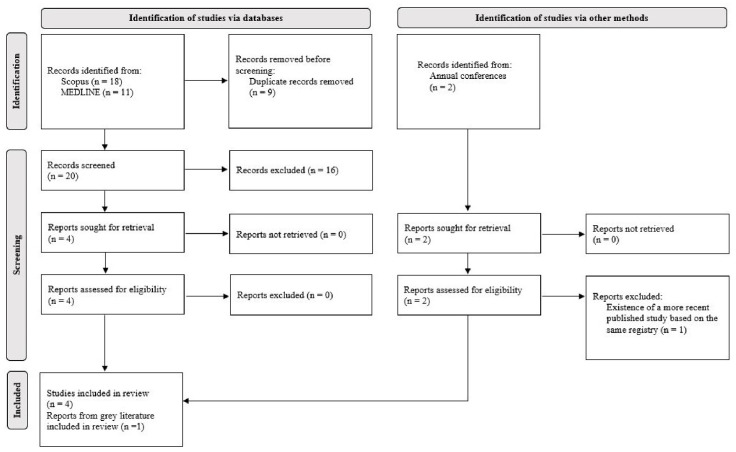
Flow diagram of study selection.

The literature search of the MEDLINE and Scopus databases yielded 29 records. After the removal of duplicates, 20 articles remained for title and abstract screening. Ultimately, four studies were reviewed and selected following full-text assessment. An additional two records were identified through gray literature searches, of which one was excluded due to the availability of a more recent peer-reviewed publication based on the same registry. Thus, a total of five studies were included in the systematic review and meta-analysis. The study selection flow diagram is presented in Figure 1. The main characteristics of the included studies are summarized in Table 1. All were observational studies, comprising two prospective cohort studies, two retrospective cohort studies, and one cross-sectional study [13,14,15,17,20]. The studies included a total of 1991 participants, with individual sample sizes ranging from 40 to 1115. The mean age of participants across studies ranged from 41 to 56 years, and the proportion of female participants varied between 47% and 83%.

### 3.2. Methodological Quality of Included Studies

**Figure 2:**
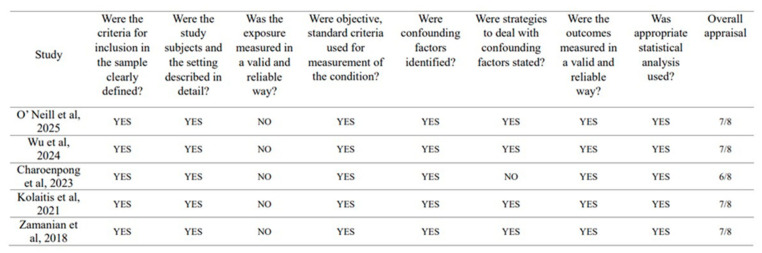
JBI Critical Appraisal Checklist for Analytical Cross-Sectional Studies [13,14,15,17,20].

All five studies clearly defined their inclusion criteria, described the study population and setting in detail, and applied appropriate statistical analyses. Standardized criteria were used for PAH diagnosis, and outcomes were measured using validated functional and hemodynamic assessments. Exposure measurement was considered unreliable in all studies, due to the absence of clearly defined and standardized criteria for quantifying sufficient methamphetamine exposure for the diagnosis of Meth-APAH. Confounding factors were identified in all studies, but strategies to address them were implemented in four [13,14,17,20]. Most studies received positive ratings in seven out of eight domains, indicating high methodological quality [13,14,17,20], while one study scored six positive ratings and was therefore considered to be of moderate quality [15]. Detailed assessments of study quality are presented in diagrammatic form in Figure 2.

### 3.3. Primary Outcomes

**Figure 3:**
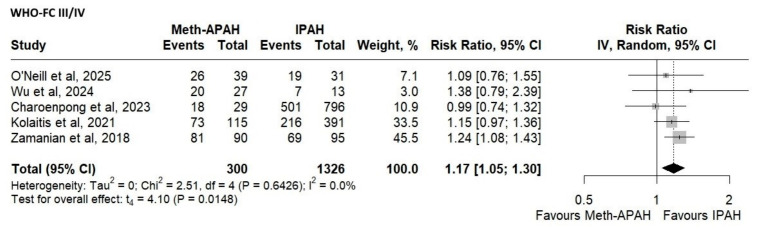
Forest plot of the relative risk of classification as WHO-FC III/IV. The size of the squares represents the weight assigned to each individual study, while the horizontal lines indicate the 95% confidence intervals. The diamond represents the overall pooled estimate. CI: confidence interval, IPAH: idiopathic pulmonary arterial hypertension, Meth-APAH: methamphetamine-associated pulmonary arterial hypertension [13,14,15,17,20].

Five studies [13,14,15,17,20] provided data on the proportion of patients classified as WHO-FC III/IV. Patients with Meth-APAH had a significantly higher relative risk of being classified as WHO-FC III/IV compared to those with IPAH (RR 1.17, 95% CI 1.05–1.30, *p* = 0.01, I^2^ = 0.0%) (Figure 3).

**Figure 4:**
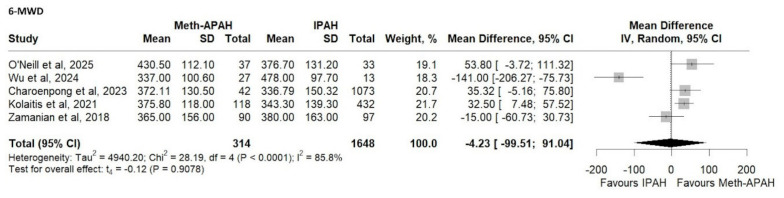
Forest plot of the 6-MWD comparison. The size of the squares represents the weight assigned to each individual study, while the horizontal lines indicate the 95% confidence intervals. The diamond represents the overall pooled estimate. CI: confidence interval, IPAH: idiopathic pulmonary arterial hypertension, Meth-APAH: methamphetamine-associated pulmonary arterial hypertension [13,14,15,17,20].

The 6-MWD was also assessed in the same five studies. No statistically significant difference was observed between Meth-APAH and IPAH (MD −4.23, 95% CI −99.51–91.04, *p* = 0.9, I^2^ = 85.8%), although substantial heterogeneity was present. Four studies [13,14,15,20] reported data on mPAP (Figure 4).

**Figure 5:**
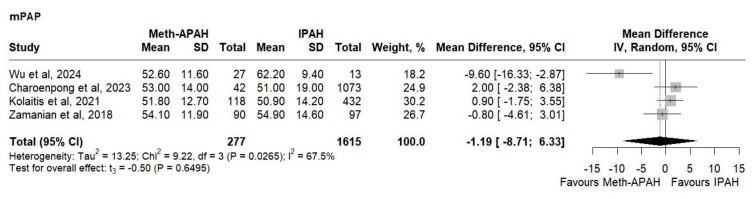
Forest plot of the mPAP comparison. The size of the squares represents the weight assigned to each individual study, while the horizontal lines indicate the 95% confidence intervals. The diamond represents the overall pooled estimate. CI: confidence interval, IPAH: idiopathic pulmonary arterial hypertension, Meth-APAH: methamphetamine-associated pulmonary arterial hypertension [13,14,15,20].

The pooled analysis showed no significant difference in mPAP between Meth-APAH and IPAH patients (MD −1.19, 95% CI −8.71–6.33, *p* = 0.65, I^2^ = 67.5%) [13,14,15,20] (Figure 5).

### 3.4. Secondary Outcomes

**Figure 6:**
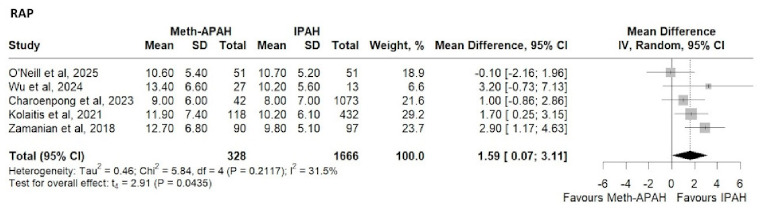
Forest plot of the RAP comparison. The size of the squares represents the weight assigned to each individual study, while the horizontal lines indicate the 95% confidence intervals. The diamond represents the overall pooled estimate. CI: confidence interval, IPAH: idiopathic pulmonary arterial hypertension, Meth-APAH: methamphetamine-associated pulmonary arterial hypertension [13,14,15,17,20].

Five studies [13,14,15,17,20] reported data on RAP. Patients with Meth-APAH exhibited significantly higher RAP values compared to those with IPAH (MD 1.59, 95% CI 0.07–3.11, *p* = 0.04, I^2^ = 31.5%) (Figure 6).

**Figure 7:**
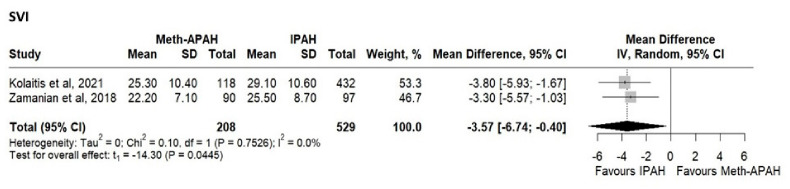
Forest plot of the SVI comparison. The size of the squares represents the weight assigned to each individual study, while the horizontal lines indicate the 95% confidence intervals. The diamond represents the overall pooled estimate. CI: confidence interval, IPAH: idiopathic pulmonary arterial hypertension, Meth-APAH: methamphetamine-associated pulmonary arterial hypertension [13,14].

Based on data from two studies [13,14], patients with Meth-APAH had significantly lower SVI values compared to those with IPAH (MD −3.57, 95% CI −6.74–−0.40, *p* = 0.04, I^2^ = 0.0%) (Figure 7).

**Figure 8:**
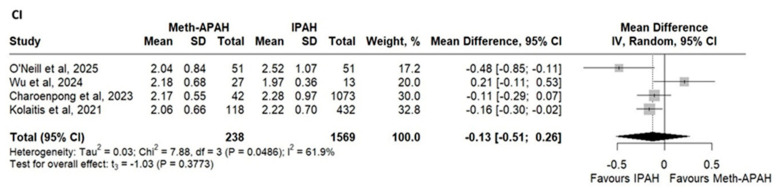
Forest plot of the cardiac index comparison. The size of the squares represents the weight assigned to each individual study, while the horizontal lines indicate the 95% confidence intervals. The diamond represents the overall pooled estimate. CI: confidence interval, IPAH: idiopathic pulmonary arterial hypertension, Meth-APAH: methamphetamine-associated pulmonary arterial hypertension [14,15,17,20].

Four studies provided data on CI [14,15,17,20]. No significant difference was observed between the Meth-APAH and IPAH groups (MD −0.13, 95% CI −0.51–0.26, *p* = 0.38, I^2^ = 61.9%) (Figure 8).

**Figure 9:**
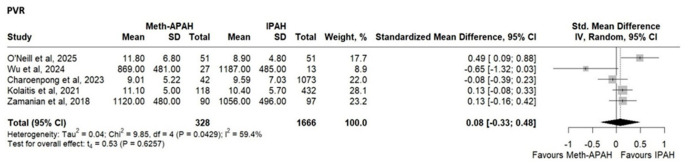
Forest plot of the PVR comparison. The size of the squares represents the weight assigned to each individual study, while the horizontal lines indicate the 95% confidence intervals. The diamond represents the overall pooled estimate. CI: confidence interval, IPAH: idiopathic pulmonary arterial hypertension, Meth-APAH: methamphetamine-associated pulmonary arterial hypertension [13,14,15,17,20].

**Figure 10:**
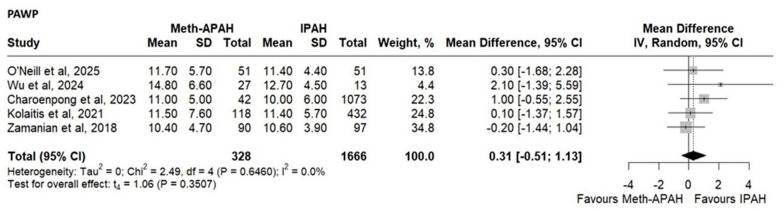
Forest plot of the PAWP comparison. The size of the squares represents the weight assigned to each individual study, while the horizontal lines indicate the 95% confidence intervals. The diamond represents the overall pooled estimate. CI: confidence interval, IPAH: idiopathic pulmonary arterial hypertension, Meth-APAH: methamphetamine-associated pulmonary arterial hypertension [13,14,15,17,20].

**Figure 11:**
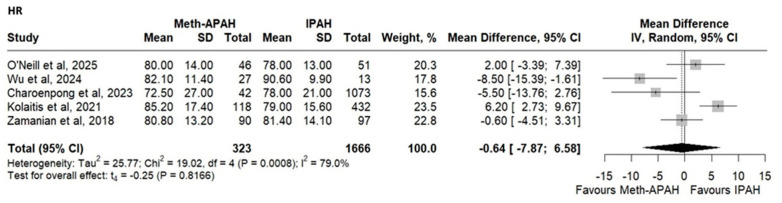
Forest plot of the HR comparison. The size of the squares represents the weight assigned to each individual study, while the horizontal lines indicate the 95% confidence intervals. The diamond represents the overall pooled estimate. CI: confidence interval, IPAH: idiopathic pulmonary arterial hypertension, Meth-APAH: methamphetamine-associated pulmonary arterial hypertension [13,14,15,17,20].

Data from five studies [13,14,15,17,20] showed no significant differences between Meth-APAH and IPAH groups in PVR (SMD 0.08, 95% CI −0.33–0.48, *p* = 0.63, I^2^ = 59.4%), PAWP (MD 0.31, 95% CI −0.51–1.13, *p* = 0.35, I^2^ = 0.0%), or HR (MD −0.64, 95% CI −7.87–6.58, *p* = 0.82, I^2^ = 79%) (Figure 9, Figure 10 and Figure 11).

### 3.5. Sensitivity Analyses

Multiple sensitivity analyses were conducted to assess the robustness of the main findings. Specifically, three separate analyses were performed: exclusion of the matched cohort study [17], exclusion of the conference abstract [20] and exclusion of the study with significant imbalance in the sample sizes of the Meth-APAH and IPAH groups [15]. Statistical significance for the WHO-FC III/IV outcome was retained across all sensitivity analyses. In contrast, the exclusion of the conference abstract and the study with sample size imbalance led to a loss of statistical significance for the difference in RAP (MD 1.47, 95% CI −0.45–3.39, *p* = 0.09 and MD 1.76, 95% CI −0.47–3.99, *p* = 0.09, respectively). Outcomes that were non-significant in the main analysis remained non-significant across all sensitivity analyses. Heterogeneity showed minor fluctuations between the main and sensitivity analyses, with the most notable change observed in the mPAP outcome, where I^2^ decreased from 67.5% to 0.0% following the exclusion of the conference abstract. SVI was assessed in only two studies and was therefore not included in the sensitivity analyses. A detailed summary of the sensitivity analysis results is provided in Table 2.

## 4. Discussion

The aim of this systematic review and meta-analysis was to compare functional and hemodynamic parameters between patients with Meth-APAH and IPAH. According to the study findings, patients with Meth-APAH had a significantly higher relative risk of being classified as WHO-FC III/IV, and this was the steadiest finding retained across all analyses. Furthermore, patients with Meth-APAH presented with higher RAP and reduced SVI compared to those with IPAH, but these differences were modest. No statistically significant differences were observed between the two groups in terms of 6-MWD, mPAP, CI, PVR, PAWP, and HR parameters. Moreover, values of 6-MWD, PVR, CI, and PAWP tended to be worse, while those of mPAP and HR tended to be more favorable in patients with Meth-APAH, compared to those in patients with IPAH. Of the above parameters, WHO-FC III/IV, 6-MWD, RAP, CI, PVR, and SVI are generally considered as predictors of prognosis and are widely used in risk stratification and the selection of appropriate therapeutic strategies for patients with PAH [21,22]. Nevertheless, the differences which were noted in the current meta-analysis are not in the range of values that have been associated with poor outcome. Thus, their clinical and prognostic significance remains uncertain.

As far as the RAP outcome is regarded, certain limitations need to be taken under consideration. In the sensitivity analyses, the exclusion of the conference abstract [20] and the study with a substantial sample size imbalance [15] led to the loss of statistical significance for the RAP outcome, confirming that these studies exerted a notable influence on the pooled estimate. Conversely, statistical significance for the WHO-FC III/IV outcome was maintained across all sensitivity analyses, indicating the robustness of this result. Therefore, the RAP difference should be interpreted with caution, as it appears to be less robust and more sensitive to the inclusion of specific studies than the WHO-FC III/IV outcome. Parameters that did not reach statistical significance in the primary analysis consistently remained non-significant in all sensitivity analyses, thereby reinforcing the overall reliability and stability of the results.

Another point that should be stressed is that heterogeneity across the analyses of different parameters ranged from negligible to high. The most notable reduction in heterogeneity between the main and sensitivity analyses was observed following the exclusion of the conference abstract by Wu et al. [20] in the estimation of mPAP, suggesting a disproportionate contribution of that study to the overall variability in the results. Wu et al. also reported several functional and hemodynamic parameters that differed in direction or magnitude from those of the remaining studies [20]. These discrepancies may be attributable to the limited methodological detail inherent to the conference abstract format, the small sample size, the single-center design, and the lack of information regarding patient selection, disease duration, and treatment exposure. Nevertheless, the study was retained in the primary analysis because of the limited published evidence on Meth-APAH and to reduce reporting bias. Furthermore, sensitivity analyses demonstrated that its exclusion did not materially alter the overall conclusions, despite its contribution to between-study heterogeneity. Overall, heterogeneity observed across all analyses is likely multifactorial, reflecting clinical and methodological differences between studies, including variability in study design, baseline patient characteristics and disease severity, methamphetamine exposure definitions, treatment exposure at assessment, and disease stage at enrolment [23].

Besides heterogeneity of included studies, this systematic review and meta-analysis represents the first comprehensive attempt to compare functional and hemodynamic parameters between patients with Meth-APAH and those with IPAH, in an attempt to reveal any phenotype and prognostic differences. Strengths of this study include adherence to the PRISMA guidelines, inclusion of both published and unpublished studies, the conduct of sensitivity analyses to assess the robustness of findings, and the thorough evaluation of study quality and risk of bias among the included studies.

As expected, this systematic review and meta-analysis is not without limitations. Although most of the included studies were cohort designs, the meta-analysis relied solely on baseline measurements, effectively making the data cross-sectional. Accordingly, study quality was assessed using a cross-sectional tool, which may not fully capture biases inherent to longitudinal cohort designs [19]. Also, the number of eligible studies was small and their design varied. Importantly, the absence of clearly defined and standardized criteria for quantifying adequate methamphetamine exposure for the diagnosis of Meth-APAH, together with reliance on heterogeneous diagnostic approaches—including self-reported clinical history, structured questionnaires, and inconsistent use of urine toxicology screening—resulted in substantial variation in exposure assessment across studies [13,14,15,17,20]. This likely introduced exposure misclassification bias and reduced the certainty of the pooled estimates.

Moreover, given the observational nature of the included studies, residual confounding remains an important limitation of this meta-analysis. Although some studies reported and adjusted for variables such as ethnicity, socioeconomic status, and comorbidities [13,14,15], the availability and consistency of these data varied across studies. Other important confounders, including concurrent substance use, treatment adherence, and disease duration, were incompletely reported or not consistently accounted for [13,14]. Also, some of the included studies exhibited discrepancies between the total number of participants and the number of patients with available data for specific outcomes, without explicitly reporting the distribution of data across the comparison groups [13,14,15]. In these cases, the total sample size was used in the statistical synthesis. Moreover, this meta-analysis relied on unadjusted data, as most studies did not report adjusted estimates. Where adjusted analyses were available, they tended to attenuate between-group differences and to eliminate statistical significance, indicating that the pooled estimates may be affected by confounding and should be interpreted as unadjusted associations [24]. Furthermore, due to the exclusive origin of the studies from the United States, the generalizability of the results may be limited. Formal assessment of publication bias using funnel plots was not performed because the small number of included studies precluded meaningful evaluation. Nevertheless, publication bias cannot be excluded and should be considered when interpreting the findings. Hence, gray literature was included in the search strategy to minimize the potential impact of publication bias by identifying relevant non-peer-reviewed or unpublished studies.

The findings of this meta-analysis suggest that Meth-APAH could be associated with a more adverse functional and hemodynamic profile compared to IPAH, although differences between the two disease entities are generally modest. The limited functionality according to WHO-FC and increased RAP, as well as the lower SVI, could be supported by the hypothesis of methamphetamine-induced myocardial toxicity and dysfunction, demonstrating the need for more immediate and thorough management strategies [25]. Methamphetamine exerts direct cardiotoxic effects through multiple interconnected mechanisms including increased production of reactive oxygen species, depletion of various endogenous antioxidants, impairment of mitochondrial energy metabolism, and activation of pro-inflammatory and pro-apoptotic signaling pathways. These processes have been associated with myocardial fiber hypertrophy, myocardial infarction, interstitial and perivascular fibrosis, atherosclerosis, congestion and focal degeneration or necrosis, leading to impaired cardiac function [26]. It is therefore necessary to investigate the potential effect of methamphetamine on left heart function, as it may contribute to increased left atrial pressures and the development of post-capillary PH and, consequently, to hemodynamic changes in the right heart [27]. The finding of increased RAP during RHC is consistent with the echocardiographic findings of existing studies, according to which patients with Meth-APAH are more likely to present with dilatation and dysfunction of the right heart chambers, indicative of more severe right ventricular overload and associated with a poorer prognosis [3,17]. However, RHC provides a static assessment under resting conditions and may not fully capture abnormalities in right ventricular reserve, ventriculo-arterial coupling, or exercise-induced hemodynamic responses that become evident during physical exertion [28,29].

Overall, these observations suggest that the discrepancy between worse functional status in Meth-APAH and relatively similar hemodynamic parameters compared with IPAH is unlikely to be explained by pulmonary vascular hemodynamics or cardiac dysfunction alone. Chronic methamphetamine exposure has been associated with neurotoxicity and autonomic dysregulation, which may impair physical activity [30]. In addition, methamphetamine withdrawal is characterized by depression, anxiety, cognitive impairment, sleep disturbance, fatigue, and musculoskeletal pain, with some symptoms potentially persisting beyond cessation of use and further limiting physical functioning [31]. Malnutrition, psychiatric and medical comorbidities, and reduced adherence to PAH-specific therapy may also contribute to poorer functional status [13]. Collectively, these factors provide a plausible explanation for the consistently higher prevalence of WHO functional class III/IV observed in patients with Meth-APAH, despite only modest differences in conventional hemodynamic parameters.

Due to the absence of statistically significant differences between the other hemodynamic parameters, it remains unclear whether specialized therapeutic interventions for Meth-APAH are required. The more favorable but not statistically significant mPAP and HR values observed in patients with Meth-APAH may indicate differences in pathophysiology between the two groups but should be interpreted with caution.

The above findings are largely consistent with previous single-center and multicenter studies. The majority of these studies did not detect statistical significance between patients with Meth-APAH and IPAH for the parameters 6-MWD, mPAP, CI, PVR, PAWP, and HR [13,14,15,17,20]. The significant difference in RAP was driven by two of the included studies [13,14], while the statistically significant increased risk of being classified as WHO-FC III/IV was primarily attributable to the findings of a single study [13]. Moreover, the lack of sufficient published data on clinical important outcomes, such as morbidity and Meth-APAH-related mortality and/or hospitalizations, further limits the differentiation between Meth-APAH and IPAH, in terms of clinical presentation and prognosis.

## 5. Conclusions

This systematic review and meta-analysis represents the first comprehensive attempt to compare functional and hemodynamic parameters between patients with Meth-APAH and those with IPAH. The findings suggest that Meth-APAH may be associated with a more adverse profile. Given the limited number and heterogeneity of available studies, and also the scarce published data regarding outcomes with high prognostic significance, there is a clear need for further research in this field. Future research should focus on establishing standardized criteria for quantifying methamphetamine exposure, conducting large-scale prospective studies, elucidating the underlying disease mechanisms, and comparing therapeutic responses. Such efforts will be essential to achieving a more comprehensive, evidence-based understanding of Meth-APAH and, ultimately, to improving patient management.

## Figures and Tables

**Table 1:** Characteristics of included studies.

Characteristics	O’Neill et al. (2025) [17]	Wu et al. (2024) [20]	Charoenpong et al. (2023) [15]	Kolaitis et al. (2021) [14]	Zamanian et al. (2018) [13]
Country	USA	USA	USA	USA	USA
Journal	Echocardiography	American Journal of Respiratory and Critical Care Medicine (abstract)	BMJ Open Respiratory Research	Annals of the American Thoracic Society	American Journal of Respiratory and Critical Care Medicine
Study design	Retrospective cohort (matched for mPAP)	Retrospective cohort	Cross-sectional	Prospective cohort	Prospective cohort
Setting	Single-center/University of Utah Pulmonary Hypertension Clinic	Single-center/Los Angeles pulmonary hypertension center	Multicenter/PAH Biobank	Multicenter/PHAR Registry	Single-center/Stanford Adult Pulmonary Hypertension Program
Duration	2020–2021	2015–2022	2012–unspecified	2015–2022	2003–2015
Eligibility criteria	Patients with a diagnosis of PAH confirmed by RHC (Meth-APAH and IPAH subgroups)	Adult patients with a confirmed diagnosis of PAH (Meth-APAH and IPAH subgroups)	Patients with a diagnosis of PAH confirmed by RHC (Meth-APAH and IPAH subgroups)	Adult patients with a confirmed diagnosis of PAH (Meth-APAH and IPAH subgroups)	Adult patients with a confirmed diagnosis of PAH (Meth-APAH and IPAH subgroups)
PAH subgroups	- Meth-APAH: significant methamphetamine exposure by history or structured questionnaire - IPAH: negative methamphetamine exposure history and no other identifiable causes of PAH	- Meth-APAH: documented methamphetamine use - IPAH: negative methamphetamine exposure history	- Meth-APAH: documented methamphetamine use - IPAH: negative methamphetamine exposure history	- Meth-APAH: methamphetamine exposure by structured questionnaire - IPAH: negative methamphetamine exposure history	- Meth-APAH: significant methamphetamine exposure by structured questionnaire (>3 episodes per week for >3 months) and UTS for active use - IPAH: negative methamphetamine exposure history and UTS
Exclusion criteria	Not mentioned	Left-sided heart disease, HIV, emphysema, prior fenfluramine/phentermine use	Not mentioned	Not mentioned	Significant obstructive or restrictive defects/left ventricular and valvular disease, chronic thromboembolic disease, concomitant anorexigen and nonamphetamine illicit substance use, HIV, other causes of PH
Participants (Meth-APAH/IPAH)					
Mean follow-up time	54.8 months	Not mentioned	-	13.4 months	47.2 months
Sample size	51/51 *n* = 102	27/13 *n* = 40	42/1073 *n* = 1115	118/432 *n* = 550	90/97 *n* = 187
Age, years ± SD	46.6 ± 10.5/55.5 ± 14.7	42.6 ± 8.8/41.3 ± 17.2	43.6 ± 10.3/48.7 ± 25.9	47.5 ± 9.2/55.5 ± 17.2	44.5 ± 7.3/ 45.2 ± 16.4
Sex, female %	47/80	66.7/78.6	69.1/78.4	62.7/76.4	63.3/82.5
Outcomes (Meth-APAH/IPAH)					
WHO-FC III/IV, *n* (%)	26 (66)/19 (61)	20 (74.1)/7 (53.4)	18 (62.94)/501 (62.06)	73 (64)/216 (55)	81 (90)/69 (71.1)
6-MWD, m ± SD	430.5 ± 112/376.7 ± 131.2	337 ± 100.6/478 ± 97.7	372.11 ± 130.5/336.79 ± 150.32	375.8 ± 118/343.3 ± 139.3	365 ± 156/380 ± 163
mPAP, mmHg ± SD	49.5 ± 13.3/49.5 ± 13.2	52.6 ± 11.6/62.2 ± 9.4	53 ± 14/51 ± 19	51.8 ± 12.7/50.9 ± 14.2	54.1 ± 11.9/54.9 ± 141.6
CI, L/min/m^2^ ± SD	2.04 ± 0.84/2.52 ± 1.07	2.18 ± 0.68/1.97 ± 0.36	2.17 ± 0.55/2.28 ± 0.97	2.06 ± 0.66/0.22 ± 0.7	-
RAP, mmHg ± SD	10.6 ± 5.4/10.7 ± 5.2	13.4 ± 6.6/10.2 ± 5.6	9 ± 6/8 ± 7	11.9 ± 7.4/10.2 ± 6.1	12.7 ± 6.8/9.8 ± 5.1
PVR, WU ή dyn/sec/cm^−5^ ±SD	11.8 ± 6.8/8.9 ± 4.8	869 ± 481/1187 ± 485	9.01 ± 5.22/9.59 ± 7.03	11.1 ± 5/10.4 ± 5.7	1120 ± 480/1056 ± 496
SVI, ml/beat/m^2^ ± SD	-	-	-	25.3 ± 10.4/29.1 ± 10.6	22.2 ± 7.1/25.5 ± 8.7
PAWP, mmHg ± SD	11.7 ± 5.7/11.4 ± 4.4	14.8 ± 6.6/12.7 ± 4.5	11 ± 5/10 ± 6	11.5 ± 7.6/11.4 ± 5.7	10.4 ± 4.7/10.6 ± 3.9
HR, beats/min ± SD	80 ± 14/78 ± 13	82.1 ± 11.4/90.6 ± 9.9	72.5 ± 27/78 ± 21	85.2 ± 17.4/79 ± 15.6	80.8 ± 13.2/81.4 ± 14.1

6-MWD: six-minute walk distance, CI: cardiac index, HIV: human immunodeficiency virus, HR: heart rate, IPAH: idiopathic pulmonary arterial hypertension, Meth-APAH: methamphetamine-associated pulmonary arterial hypertension, mPAP: mean pulmonary arterial pressure, PAH: pulmonary arterial hypertension, PAWP: pulmonary artery wedge pressure, PH: pulmonary hypertension, PVR: pulmonary vascular resistance, RAP: right atrial pressure, RHC: right heart catheterization, SD: standard deviation, SVI: stroke volume index, UTS: urine toxicology screening, WHO-FC: World Health Organization functional classification, WU: Wood units.

**Table 2:** Summary table of main and sensitivity analyses result.

Outcomes	Main Analysis RR/MD/SMD (CI 95%)	Exclusion of Matched Study (O’Neill et al.) [17]	Exclusion of Conference Abstract (Wu et al.) [20]	Exclusion of Study with Sample Size Imbalance (Charoenpong et al.) [15]
WHO-FC III/IV	1.17 (1.05–1.30), I^2^ 0%, *p* = 0.01	1.18 (1.02–1.36), I^2^ 0%, *p* = 0.04	1.17 (1.02–1.33), I^2^ 0%, *p* = 0.04	1.20 (1.09–1.31), I^2^ 0%, *p* = 0.01
6-MWD	−4.23 (−99.51–91.04), I^2^ 85.8%, *p* = 0.9	−18.39 (−146.6–109.84), I^2^ 88.6%, *p* = 0.68	26.91 (−12.13–65.96), I^2^ 32.1%, *p* = 0.12	−15.11 (−151.15–120.92), I^2^ 88.9%, *p* = 0.75
mPAP	−1.19 (−8.71–6.33), I^2^ 67.5%, *p* = 0.65	-	0.67 (2.29–3.36), I^2^ 0%, *p* = 0.43	−2.47 (−15.65–10.71), I^2^ 75.3%, *p* = 0.50
CI	−0.13 (−0.13–0.26), I^2^ 61.9%, *p* = 0.38	−0.07 (−0.49–0.34), I^2^ 53.4%, *p* = 0.53	−0.17 (−0.45–0.12), I^2^ 35.7%, *p* = 0.13	−0.14 (−0.95–0.68), I^2^ 74.3%, *p* = 0.55
RAP	1.59 (0.07–3.11), I^2^ 31.5%, *p* = 0.04	1.95 (0.54–3.37), I^2^ 0%, *p* = 0.02	1.47 (−0.45–3.39), I^2^ 42%, *p* = 0.09	1.76 (−0.47–3.99), I^2^ 43.6%, *p* = 0.09
PVR	0.08 (−0.33–0.48), I^2^ 59.4%, *p* = 0.63	0.04 (−0.29–0.37), I^2^ 46.4, *p* = 0.26	0.13 (−0.18–0.45), I^2^ 40.3%, *p* = 0.26	0.10 (−0.52–0.73), I^2^ 63.4%, *p* = 0.63
SVI	−3.57 (−6.74–−0.4), I^2^ 0%, *p* = 0.04	-	-	-
PAWP	0.31 (−0.51–1.13), I^2^ 0%, *p* = 0.35	0.31 (−0.85–1.48), I^2^ 0%, *p* = 0.46	0.23 (−0.61–1.07), I^2^ 0%, *p* = 0.45	0.11 (−0.85–1.08), I^2^ 0%, *p* = 0.73
HR	−0.64 (−7.87–6.58), I^2^ 79%, *p* = 0.82	−1.49 (−11.83–8.86), I^2^ 84.2%, *p* = 0.68	1.3 (−6.06–8.66), I^2^ 71.2%, *p* = 0.61	0.22 (−9.33–9.78), I^2^ 81.5%, *p* = 0.95

6-MWD: six-minute walk distance, CI: cardiac index, HR: heart rate, MD: mean difference, mPAP: mean pulmonary arterial pressure, PAWP: pulmonary artery wedge pressure, PVR: pulmonary vascular resistance, RAP: right atrial pressure, RR: risk ratio, SMD: standardized mean difference, SVI: stroke volume index, WHO-FC III/IV: World Health Organization functional classification.

## Data Availability

Data is contained within the article or Supplementary Material.

## Supplementary Materials

The following supporting information can be downloaded at: https://www.mdpi.com/article/10.3390/healthcare14142089/s1, S1. Search strategy; S2. Forest plots of sensitivity analyses.
