# Supplementary material for: Comparison of Functional and Hemodynamic Parameters Between Methamphetamine-Associated and Idiopathic Pulmonary Arterial Hypertension: Systematic Review and Meta-Analysis
Source: Healthcare (Basel). 2026 Jul 13;14(14):2089. doi: 10.3390/healthcare14142089 (PMC13411680; doi:10.3390/healthcare14142089)
Supplement: Supplementary file 1 [file healthcare-14-02089-s001.zip › healthcare-4362572-supplementary.pdf]

Supplementary material

S1. Search strategy

i. MEDLINE via PubMed

(("Methamphetamine"[MeSH Terms] OR "methamphetamine"[All Fields] OR "methamphetamine associated"[All Fields] OR "methamphetamine-associated pulmonary arterial hypertension"[All Fields]) AND ("Pulmonary Arterial Hypertension"[MeSH Terms] OR "PAH"[All Fields])) AND ("idiopathic pulmonary arterial hypertension"[All Fields] OR "idiopathic PAH"[All Fields] OR "idiopathic pulmonary hypertension"[All Fields])

ii. Scopus

(TITLE-ABS-KEY ("methamphetamine" OR "methamphetamine-associated" OR "methamphetamine associated pulmonary arterial hypertension") AND TITLE-ABS-KEY ("pulmonary arterial hypertension" OR "PAH") AND TITLE-ABS-KEY ("idiopathic pulmonary arterial hypertension" OR "idiopathic PAH" OR "idiopathic pulmonary hypertension"))

S2. Forest plots of sensitivity analyses

i. Exclusion of matched study (O'Neill et al.)

WHO-FC III/IV

Study	Meth-APAH Events	Meth-APAH Total	IPAH Events	IPAH Total	Weight, %	Risk Ratio, 95% CI
Wu et al, 2024	20	27	7	13	3.2	1.38 [0.79; 2.39]
Charoenpong et al, 2023	18	29	501	796	11.7	0.99 [0.74; 1.32]
Kolaitis et al, 2021	73	115	216	391	36.0	1.15 [0.97; 1.36]
Zamanian et al, 2018	81	90	69	95	49.0	1.24 [1.08; 1.43]
**Total (95% CI)**		**261**		**1295**	**100.0**	**1.18 [1.02; 1.36]**

Heterogeneity: $Tau^2 = 0$; $Chi^2 = 2.33$, df = 3 (P = 0.5062); $I^2 = 0.0\%$
Test for overall effect: $t_3 = 3.68$ (P = 0.0347)

Risk Ratio
IV, Random, 95% CI

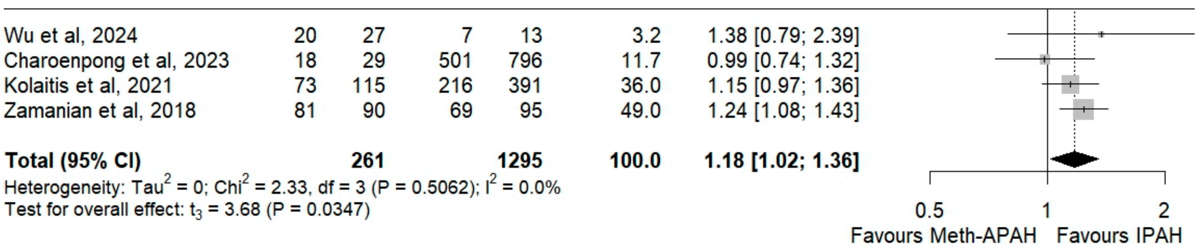

6-MWD

Study	Meth-APAH Mean	Meth-APAH SD	Meth-APAH Total	IPAH Mean	IPAH SD	IPAH Total	Weight, %	Mean Difference, 95% CI
Wu et al, 2024	337.00	100.60	27	478.00	97.70	13	22.9	-141.00 [-206.27; -75.73]
Charoenpong et al, 2023	372.11	130.50	42	336.79	150.32	1073	25.5	35.32 [-5.16; 75.80]
Kolaitis et al, 2021	375.80	118.00	118	343.30	139.30	432	26.6	32.50 [7.48; 57.52]
Zamanian et al, 2018	365.00	156.00	90	380.00	163.00	97	25.0	-15.00 [-60.73; 30.73]
**Total (95% CI)**			**277**			**1615**	**100.0**	**-18.39 [-146.62; 109.84]**

Heterogeneity: $Tau^2 = 5651.57$; $Chi^2 = 26.30$, df = 3 (P < 0.0001); $I^2 = 88.6\%$
Test for overall effect: $t_3 = -0.46$ (P = 0.6791)

Mean Difference
IV, Random, 95% CI

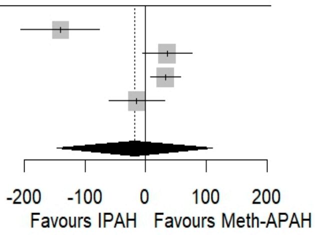

CI

Study	Meth-APAH Mean	Meth-APAH SD	Meth-APAH Total	IPAH Mean	IPAH SD	IPAH Total	Weight, %	Mean Difference, 95% CI
Wu et al, 2024	2.18	0.68	27	1.97	0.36	13	18.4	0.21 [-0.11; 0.53]
Charoenpong et al, 2023	2.17	0.55	42	2.28	0.97	1073	36.9	-0.11 [-0.29; 0.07]
Kolaitis et al, 2021	2.06	0.66	118	2.22	0.70	432	44.6	-0.16 [-0.30; -0.02]
**Total (95% CI)**			**187**			**1518**	**100.0**	**-0.07 [-0.49; 0.34]**

Heterogeneity: $Tau^2 = 0.01$; $Chi^2 = 4.29$, df = 2 (P = 0.1170); $I^2 = 53.4\%$
Test for overall effect: $t_2 = -0.76$ (P = 0.5266)

Mean Difference
IV, Random, 95% CI

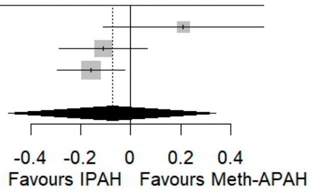

RAP

Study	Meth-APAH Mean	Meth-APAH SD	Meth-APAH Total	IPAH Mean	IPAH SD	IPAH Total	Weight, %	Mean Difference, 95% CI
Wu et al, 2024	13.40	6.60	27	10.20	5.60	13	5.6	3.20 [-0.73; 7.13]
Charoenpong et al, 2023	9.00	6.00	42	8.00	7.00	1073	24.9	1.00 [-0.86; 2.86]
Kolaitis et al, 2021	11.90	7.40	118	10.20	6.10	432	40.8	1.70 [0.25; 3.15]
Zamanian et al, 2018	12.70	6.80	90	9.80	5.10	97	28.7	2.90 [1.17; 4.63]
**Total (95% CI)**			**277**			**1615**	**100.0**	**1.95 [0.54; 3.37]**

Heterogeneity: $Tau^2 = 0$; $Chi^2 = 2.66$, df = 3 (P = 0.4478); $I^2 = 0.0\%$
Test for overall effect: $t_3 = 4.38$ (P = 0.0220)

Mean Difference
IV, Random, 95% CI

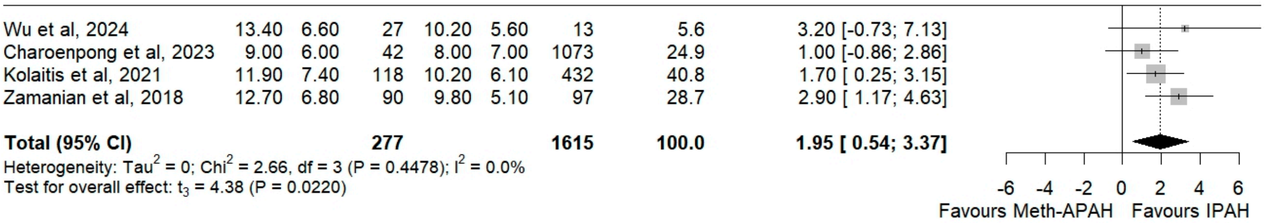

PVR

Study	Meth-APAH Mean	SD	Total	IPAH Mean	SD	Total	Weight, %	Standardized Mean Difference, 95% CI
Wu et al, 2024	869.00	481.00	27	1187.00	485.00	13	5.1	-0.65 [-1.32; 0.03]
Charoenpong et al, 2023	9.01	5.22	42	9.59	7.03	1073	22.7	-0.08 [-0.39; 0.23]
Kolaitis et al, 2021	11.10	5.00	118	10.40	5.70	432	46.5	0.13 [-0.08; 0.33]
Zamanian et al, 2018	1120.00	480.00	90	1056.00	496.00	97	25.8	0.13 [-0.16; 0.42]
**Total (95% CI)**			**277**			**1615**	**100.0**	**0.04 [-0.29; 0.37]**

Heterogeneity: $Tau^2 < 0.01$; $Chi^2 = 5.60$, df = 3 (P = 0.1329); $I^2 = 46.4\%$
Test for overall effect: $t_3 = 0.39$ (P = 0.7239)

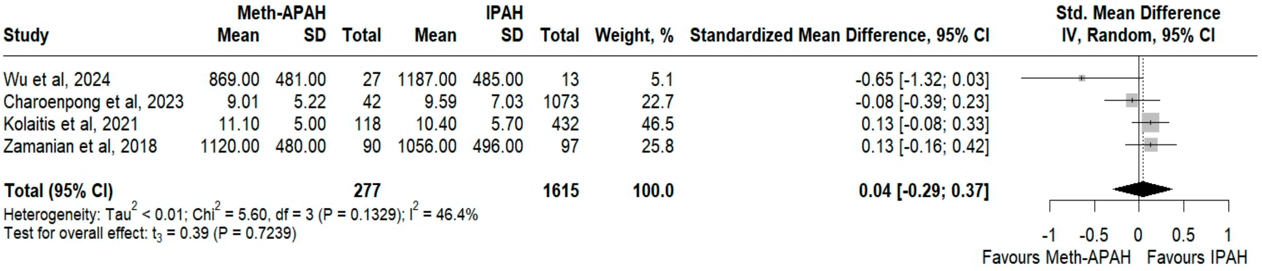

PAWP

Study	Meth-APAH Mean	SD	Total	IPAH Mean	SD	Total	Weight, %	Mean Difference, 95% CI
Wu et al, 2024	14.80	6.60	27	12.70	4.50	13	5.1	2.10 [-1.39; 5.59]
Charoenpong et al, 2023	11.00	5.00	42	10.00	6.00	1073	25.8	1.00 [-0.55; 2.55]
Kolaitis et al, 2021	11.50	7.60	118	11.40	5.70	432	28.7	0.10 [-1.37; 1.57]
Zamanian et al, 2018	10.40	4.70	90	10.60	3.90	97	40.3	-0.20 [-1.44; 1.04]
**Total (95% CI)**			**277**			**1615**	**100.0**	**0.31 [-0.85; 1.48]**

Heterogeneity: $Tau^2 = 0$; $Chi^2 = 2.49$, df = 3 (P = 0.4767); $I^2 = 0.0\%$
Test for overall effect: $t_3 = 0.85$ (P = 0.4558)

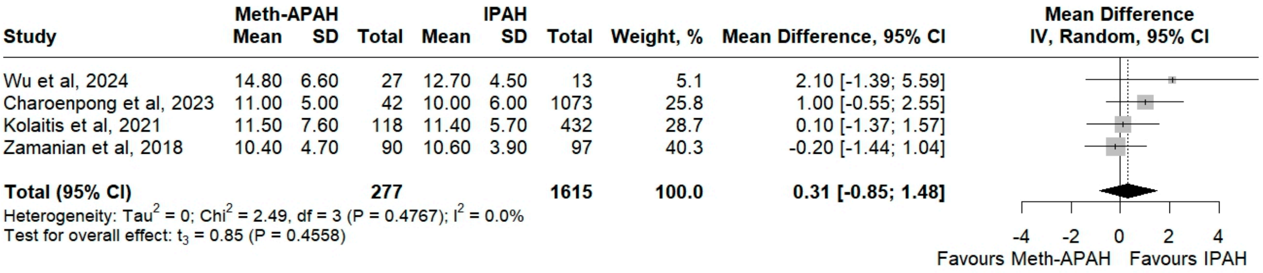

HR

Study	Meth-APAH Mean	SD	Total	IPAH Mean	SD	Total	Weight, %	Mean Difference, 95% CI
Wu et al, 2024	82.10	11.40	27	90.60	9.90	13	23.0	-8.50 [-15.39; -1.61]
Charoenpong et al, 2023	72.50	27.00	42	78.00	21.00	1073	20.6	-5.50 [-13.76; 2.76]
Kolaitis et al, 2021	85.20	17.40	118	79.00	15.60	432	28.5	6.20 [2.73; 9.67]
Zamanian et al, 2018	80.80	13.20	90	81.40	14.10	97	27.9	-0.60 [-4.51; 3.31]
**Total (95% CI)**			**277**			**1615**	**100.0**	**-1.49 [-11.83; 8.86]**

Heterogeneity: $Tau^2 = 35.19$; $Chi^2 = 18.95$, df = 3 (P = 0.0003); $I^2 = 84.2\%$
Test for overall effect: $t_3 = -0.46$ (P = 0.6783)

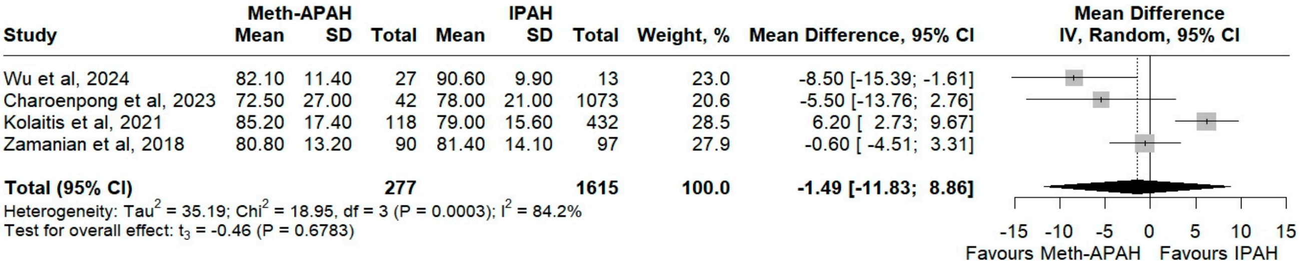

ii. Exclusion of conference abstract (Wu et al.)

WHO-FC III/IV

Study	Meth-APAH Events	Total	IPAH Events	Total	Weight, %	Risk Ratio, 95% CI
O'Neill et al, 2025	26	39	19	31	7.4	1.09 [0.76; 1.55]
Charoenpong et al, 2023	18	29	501	796	11.2	0.99 [0.74; 1.32]
Kolaitis et al, 2021	73	115	216	391	34.5	1.15 [0.97; 1.36]
Zamanian et al, 2018	81	90	69	95	46.9	1.24 [1.08; 1.43]
**Total (95% CI)**		**273**		**1313**	**100.0**	**1.17 [1.02; 1.33]**

Heterogeneity: $Tau^2 = 0$; $Chi^2 = 2.17$, df = 3 (P = 0.5372); $I^2 = 0.0\%$
Test for overall effect: $t_3 = 3.64$ (P = 0.0357)

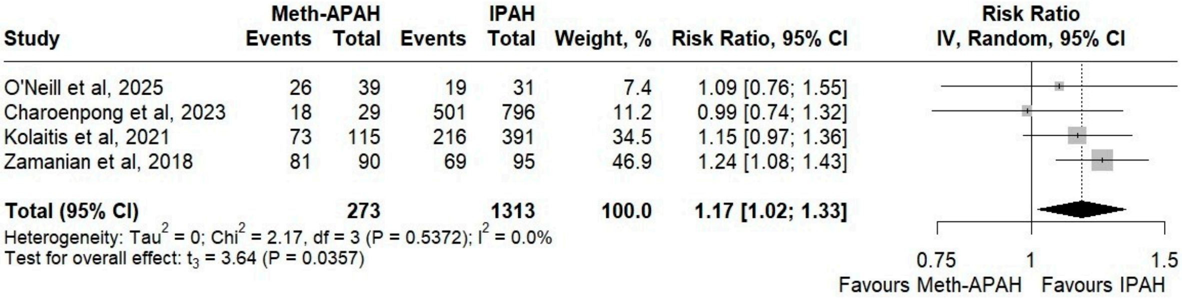

6-MWD

Study	Meth-APAH Mean	Meth-APAH SD	Meth-APAH Total	IPAH Mean	IPAH SD	IPAH Total	Weight, %	Mean Difference, 95% CI
O'Neill et al, 2025	430.50	112.10	37	376.70	131.20	33	12.6	53.80 [-3.72; 111.32]
Charoenpong et al, 2023	372.11	130.50	42	336.79	150.32	1073	23.0	35.32 [-5.16; 75.80]
Kolaitis et al, 2021	375.80	118.00	118	343.30	139.30	432	45.7	32.50 [7.48; 57.52]
Zamanian et al, 2018	365.00	156.00	90	380.00	163.00	97	18.8	-15.00 [-60.73; 30.73]
**Total (95% CI)**			**287**			**1635**	**100.0**	**26.91 [-12.13; 65.96]**

Heterogeneity: $Tau^2 = 103.69$; $Chi^2 = 4.42$, df = 3 (P = 0.2197); $I^2 = 32.1\%$
Test for overall effect: $t_3 = 2.19$ (P = 0.1159)

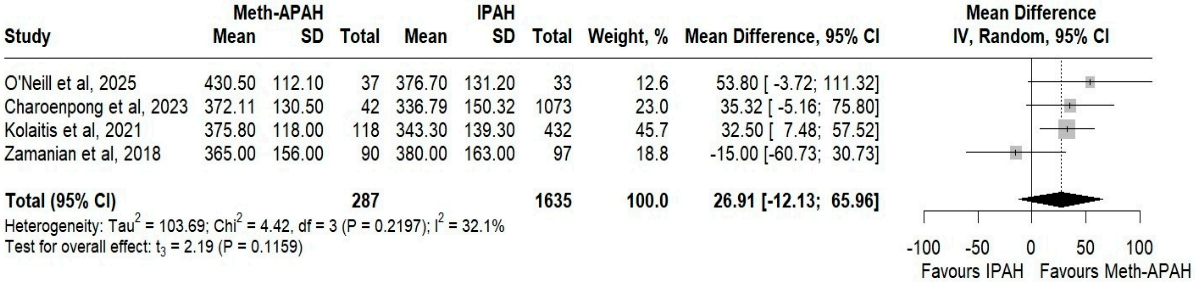

mPAP

Study	Meth-APAH Mean	Meth-APAH SD	Meth-APAH Total	IPAH Mean	IPAH SD	IPAH Total	Weight, %	Mean Difference, 95% CI
Charoenpong et al, 2023	53.00	14.00	42	51.00	19.00	1073	19.8	2.00 [-2.38; 6.38]
Kolaitis et al, 2021	51.80	12.70	118	50.90	14.20	432	54.0	0.90 [-1.75; 3.55]
Zamanian et al, 2018	54.10	11.90	90	54.90	14.60	97	26.2	-0.80 [-4.61; 3.01]
**Total (95% CI)**			**250**			**1602**	**100.0**	**0.67 [-1.28; 2.62]**

Heterogeneity: $Tau^2 = 0$; $Chi^2 = 0.96$, df = 2 (P = 0.6202); $I^2 = 0.0\%$
Test for overall effect: Z = 0.67 (P = 0.4997)

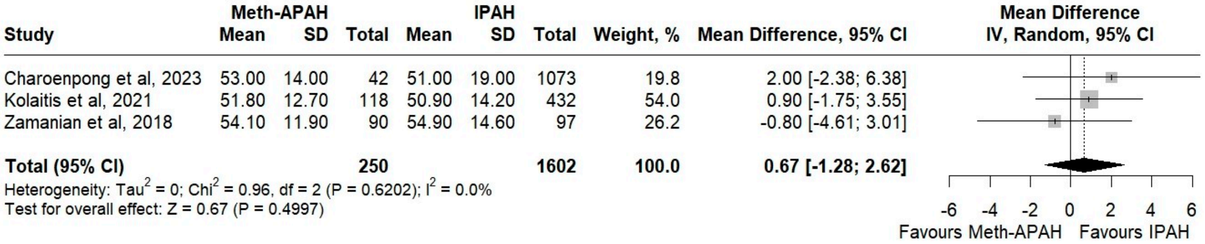

CI

Study	Meth-APAH Mean	Meth-APAH SD	Meth-APAH Total	IPAH Mean	IPAH SD	IPAH Total	Weight, %	Mean Difference, 95% CI
O'Neill et al, 2025	2.04	0.84	51	2.52	1.07	51	7.7	-0.48 [-0.85; -0.11]
Charoenpong et al, 2023	2.17	0.55	42	2.28	0.97	1073	34.5	-0.11 [-0.29; 0.07]
Kolaitis et al, 2021	2.06	0.66	118	2.22	0.70	432	57.8	-0.16 [-0.30; -0.02]
**Total (95% CI)**			**211**			**1556**	**100.0**	**-0.17 [-0.45; 0.12]**

Heterogeneity: $Tau^2 < 0.01$; $Chi^2 = 3.11$, df = 2 (P = 0.2109); $I^2 = 35.7\%$
Test for overall effect: $t_2 = -2.54$ (P = 0.1263)

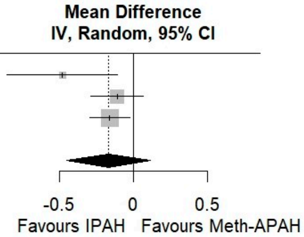

RAP

Study	Meth-APAH Mean	Meth-APAH SD	Meth-APAH Total	IPAH Mean	IPAH SD	IPAH Total	Weight, %	Mean Difference, 95% CI
O'Neill et al, 2025	10.60	5.40	51	10.70	5.20	51	20.5	-0.10 [-2.16; 1.96]
Charoenpong et al, 2023	9.00	6.00	42	8.00	7.00	1073	23.3	1.00 [-0.86; 2.86]
Kolaitis et al, 2021	11.90	7.40	118	10.20	6.10	432	30.8	1.70 [0.25; 3.15]
Zamanian et al, 2018	12.70	6.80	90	9.80	5.10	97	25.4	2.90 [1.17; 4.63]
**Total (95% CI)**			**301**			**1653**	**100.0**	**1.47 [-0.45; 3.39]**

Heterogeneity: $Tau^2 = 0.55$; $Chi^2 = 5.18$, df = 3 (P = 0.1594); $I^2 = 42.0\%$
Test for overall effect: $t_3 = 2.44$ (P = 0.0925)

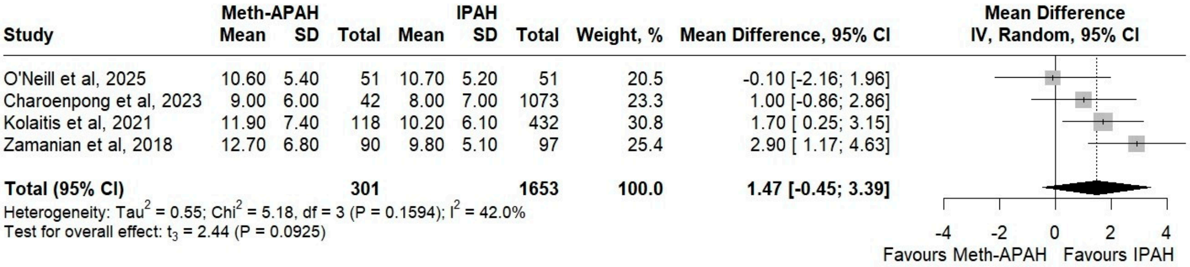

PVR

Study	Meth-APAH Mean	Meth-APAH SD	Meth-APAH Total	IPAH Mean	IPAH SD	IPAH Total	Weight, %	Standardized Mean Difference, 95% CI
O'Neill et al, 2025	11.80	6.80	51	8.90	4.80	51	14.6	0.49 [0.09; 0.88]
Charoenpong et al, 2023	9.01	5.22	42	9.59	7.03	1073	21.9	-0.08 [-0.39; 0.23]
Kolaitis et al, 2021	11.10	5.00	118	10.40	5.70	432	39.1	0.13 [-0.08; 0.33]
Zamanian et al, 2018	1120.00	480.00	90	1056.00	496.00	97	24.4	0.13 [-0.16; 0.42]
**Total (95% CI)**			**301**			**1653**	**100.0**	**0.13 [-0.18; 0.45]**

Heterogeneity: $Tau^2 < 0.01$; $Chi^2 = 5.03$, df = 3 (P = 0.1699); $I^2 = 40.3\%$
Test for overall effect: $t_3 = 1.37$ (P = 0.2636)

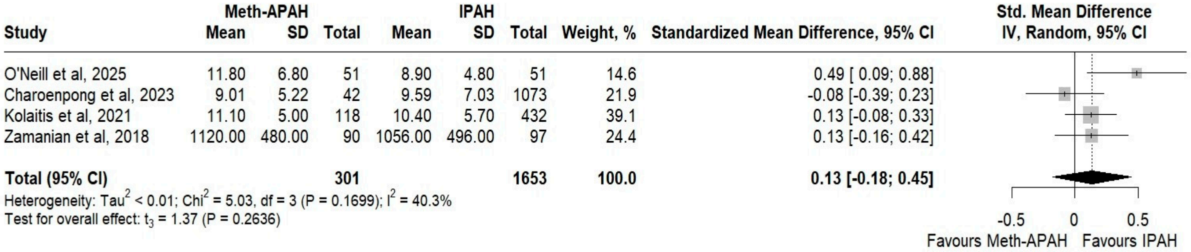

PAWP

Study	Meth-APAH Mean	SD	Total	IPAH Mean	SD	Total	Weight, %	Mean Difference, 95% CI
O'Neill et al, 2025	11.70	5.70	51	11.40	4.40	51	14.4	0.30 [-1.68; 2.28]
Charoenpong et al, 2023	11.00	5.00	42	10.00	6.00	1073	23.3	1.00 [-0.55; 2.55]
Kolaitis et al, 2021	11.50	7.60	118	11.40	5.70	432	25.9	0.10 [-1.37; 1.57]
Zamanian et al, 2018	10.40	4.70	90	10.60	3.90	97	36.4	-0.20 [-1.44; 1.04]
**Total (95% CI)**			**301**			**1653**	**100.0**	**0.23 [-0.61; 1.07]**

Heterogeneity: $Tau^2 = 0$; $Chi^2 = 1.44$, df = 3 (P = 0.6968); $I^2 = 0.0\%$
Test for overall effect: $t_3 = 0.87$ (P = 0.4505)

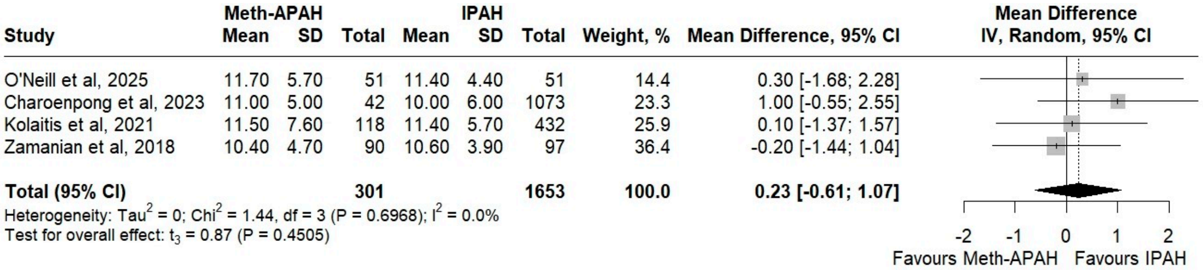

HR

Study	Meth-APAH Mean	SD	Total	IPAH Mean	SD	Total	Weight, %	Mean Difference, 95% CI
O'Neill et al, 2025	80.00	14.00	46	78.00	13.00	51	24.1	2.00 [-3.39; 7.39]
Charoenpong et al, 2023	72.50	27.00	42	78.00	21.00	1073	16.3	-5.50 [-13.76; 2.76]
Kolaitis et al, 2021	85.20	17.40	118	79.00	15.60	432	30.5	6.20 [2.73; 9.67]
Zamanian et al, 2018	80.80	13.20	90	81.40	14.10	97	29.0	-0.60 [-4.51; 3.31]
**Total (95% CI)**			**296**			**1653**	**100.0**	**1.30 [-6.06; 8.66]**

Heterogeneity: $Tau^2 = 13.76$; $Chi^2 = 10.40$, df = 3 (P = 0.0154); $I^2 = 71.2\%$
Test for overall effect: $t_3 = 0.56$ (P = 0.6130)

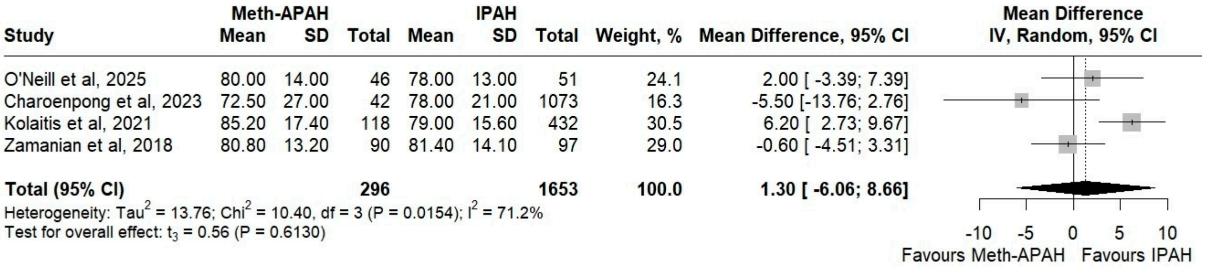

iii. Exclusion of study with sample size imbalance (Charoenpong et al.)

WHO-FC III/IV

Study	Meth-APAH Events	Total	IPAH Events	Total	Weight, %	Risk Ratio, 95% CI
O'Neill et al, 2025	26	39	19	31	8.0	1.09 [0.76; 1.55]
Wu et al, 2024	20	27	7	13	3.4	1.38 [0.79; 2.39]
Kolaitis et al, 2021	73	115	216	391	37.5	1.15 [0.97; 1.36]
Zamanian et al, 2018	81	90	69	95	51.1	1.24 [1.08; 1.43]
**Total (95% CI)**		**271**		**530**	**100.0**	**1.20 [1.09; 1.31]**

Heterogeneity: $Tau^2 = 0$; $Chi^2 = 0.99$, df = 3 (P = 0.8043); $I^2 = 0.0\%$
Test for overall effect: $t_3 = 6.06$ (P = 0.0090)

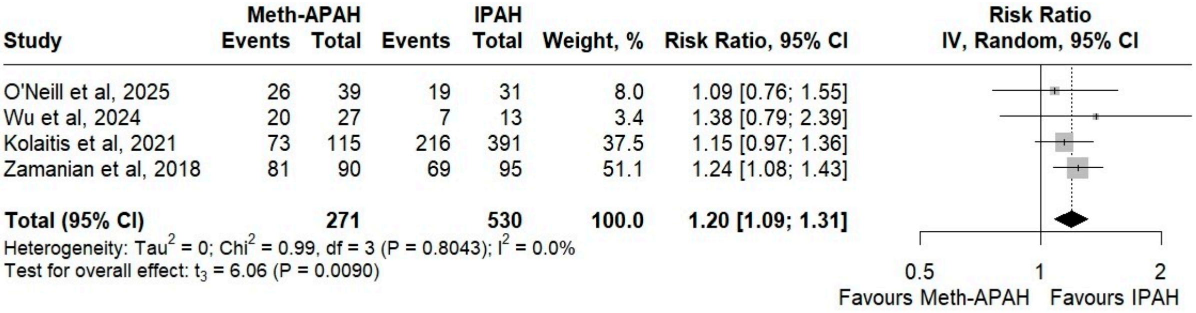

6-MWD

Study	Meth-APAH Mean	SD	Total	IPAH Mean	SD	Total	Weight, %	Mean Difference, 95% CI
O'Neill et al, 2025	430.50	112.10	37	376.70	131.20	33	24.3	53.80 [-3.72; 111.32]
Wu et al, 2024	337.00	100.60	27	478.00	97.70	13	23.5	-141.00 [-206.27; -75.73]
Kolaitis et al, 2021	375.80	118.00	118	343.30	139.30	432	26.9	32.50 [7.48; 57.52]
Zamanian et al, 2018	365.00	156.00	90	380.00	163.00	97	25.4	-15.00 [-60.73; 30.73]
**Total (95% CI)**			**272**			**575**	**100.0**	**-15.11 [-151.15; 120.92]**

Heterogeneity: $Tau^2 = 6376.20$; $Chi^2 = 27.04$, df = 3 (P < 0.0001); $I^2 = 88.9\%$
Test for overall effect: $t_3 = -0.35$ (P = 0.7471)

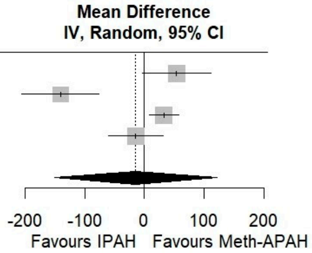

mPAP

Study	Meth-APAH Mean	Meth-APAH SD	Meth-APAH Total	IPAH Mean	IPAH SD	IPAH Total	Weight, %	Mean Difference, 95% CI
Wu et al, 2024	52.60	11.60	27	62.20	9.40	13	26.4	-9.60 [-16.33; -2.87]
Kolaitis et al, 2021	51.80	12.70	118	50.90	14.20	432	38.3	0.90 [-1.75; 3.55]
Zamanian et al, 2018	54.10	11.90	90	54.90	14.60	97	35.2	-0.80 [-4.61; 3.01]
**Total (95% CI)**			**235**			**542**	**100.0**	**-2.47 [-8.17; 3.22]**

Heterogeneity: $Tau^2 = 20.18$; $Chi^2 = 8.11$, df = 2 (P = 0.0173); $I^2 = 75.3\%$
Test for overall effect: Z = -0.85 (P = 0.3948)

Mean Difference
IV, Random, 95% CI

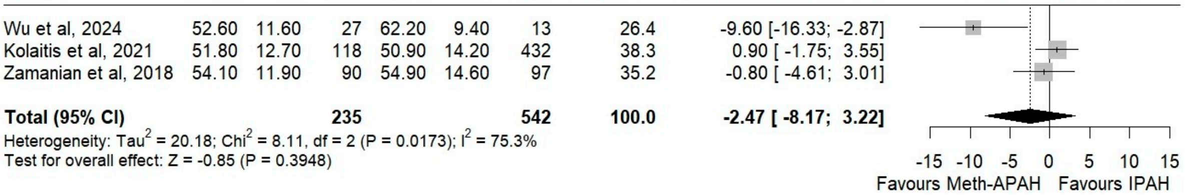

CI

Study	Meth-APAH Mean	Meth-APAH SD	Meth-APAH Total	IPAH Mean	IPAH SD	IPAH Total	Weight, %	Mean Difference, 95% CI
O'Neill et al, 2025	2.04	0.84	51	2.52	1.07	51	28.8	-0.48 [-0.85; -0.11]
Wu et al, 2024	2.18	0.68	27	1.97	0.36	13	31.4	0.21 [-0.11; 0.53]
Kolaitis et al, 2021	2.06	0.66	118	2.22	0.70	432	39.8	-0.16 [-0.30; -0.02]
**Total (95% CI)**			**196**			**496**	**100.0**	**-0.14 [-0.95; 0.68]**

Heterogeneity: $Tau^2 = 0.08$; $Chi^2 = 7.79$, df = 2 (P = 0.0204); $I^2 = 74.3\%$
Test for overall effect: $t_2 = -0.72$ (P = 0.5470)

Mean Difference
IV, Random, 95% CI

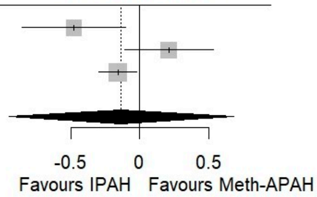

RAP

Study	Meth-APAH Mean	Meth-APAH SD	Meth-APAH Total	IPAH Mean	IPAH SD	IPAH Total	Weight, %	Mean Difference, 95% CI
O'Neill et al, 2025	10.60	5.40	51	10.70	5.20	51	25.1	-0.10 [-2.16; 1.96]
Wu et al, 2024	13.40	6.60	27	10.20	5.60	13	10.1	3.20 [-0.73; 7.13]
Kolaitis et al, 2021	11.90	7.40	118	10.20	6.10	432	34.8	1.70 [0.25; 3.15]
Zamanian et al, 2018	12.70	6.80	90	9.80	5.10	97	29.9	2.90 [1.17; 4.63]
**Total (95% CI)**			**286**			**593**	**100.0**	**1.76 [-0.47; 3.99]**

Heterogeneity: $Tau^2 = 0.88$; $Chi^2 = 5.32$, df = 3 (P = 0.1496); $I^2 = 43.6\%$
Test for overall effect: $t_3 = 2.51$ (P = 0.0866)

Mean Difference
IV, Random, 95% CI

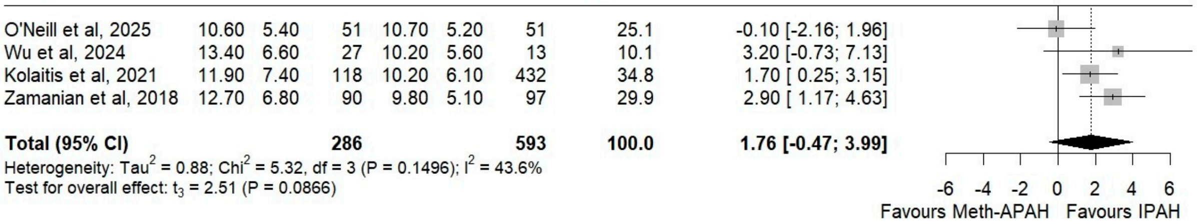

PVR

Study	Meth-APAH Mean	Meth-APAH SD	Meth-APAH Total	IPAH Mean	IPAH SD	IPAH Total	Weight, %	Standardized Mean Difference, 95% CI
O'Neill et al, 2025	11.80	6.80	51	8.90	4.80	51	24.2	0.49 [0.09; 0.88]
Wu et al, 2024	869.00	481.00	27	1187.00	485.00	13	14.3	-0.65 [-1.32; 0.03]
Kolaitis et al, 2021	11.10	5.00	118	10.40	5.70	432	32.6	0.13 [-0.08; 0.33]
Zamanian et al, 2018	1120.00	480.00	90	1056.00	496.00	97	29.0	0.13 [-0.16; 0.42]
**Total (95% CI)**			**286**			**593**	**100.0**	**0.10 [-0.52; 0.73]**

Heterogeneity: $Tau^2 = 0.07$; $Chi^2 = 8.20$, df = 3 (P = 0.0421); $I^2 = 63.4\%$
Test for overall effect: $t_3 = 0.53$ (P = 0.6320)

Std. Mean Difference
IV, Random, 95% CI

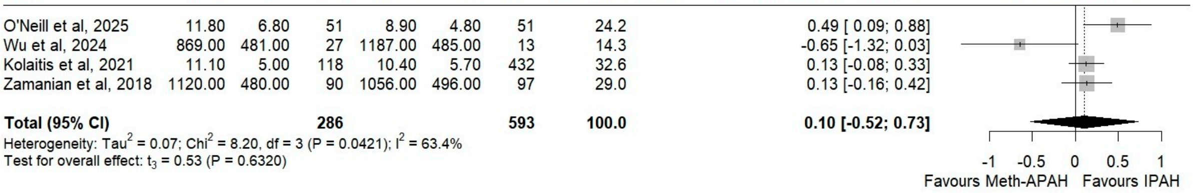

PAWP

Study	Meth-APAH Mean	Meth-APAH SD	Meth-APAH Total	IPAH Mean	IPAH SD	IPAH Total	Weight, %	Mean Difference, 95% CI
O'Neill et al, 2025	11.70	5.70	51	11.40	4.40	51	17.7	0.30 [-1.68; 2.28]
Wu et al, 2024	14.80	6.60	27	12.70	4.50	13	5.7	2.10 [-1.39; 5.59]
Kolaitis et al, 2021	11.50	7.60	118	11.40	5.70	432	31.9	0.10 [-1.37; 1.57]
Zamanian et al, 2018	10.40	4.70	90	10.60	3.90	97	44.7	-0.20 [-1.44; 1.04]
**Total (95% CI)**			**286**			**593**	**100.0**	**0.11 [-0.85; 1.08]**

Heterogeneity: $Tau^2 = 0$; $Chi^2 = 1.52$, df = 3 (P = 0.6769); $I^2 = 0.0\%$
Test for overall effect: $t_3 = 0.38$ (P = 0.7297)

Mean Difference
IV, Random, 95% CI

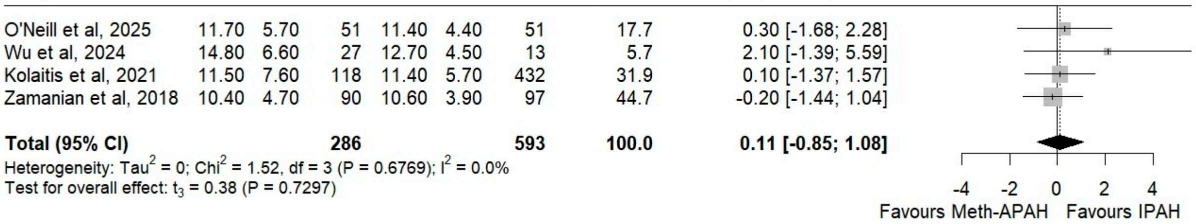

HR

Study	Meth-APAH Mean	Meth-APAH SD	Meth-APAH Total	IPAH Mean	IPAH SD	IPAH Total	Weight, %	Mean Difference, 95% CI
O'Neill et al, 2025	80.00	14.00	46	78.00	13.00	51	24.2	2.00 [-3.39; 7.39]
Wu et al, 2024	82.10	11.40	27	90.60	9.90	13	21.3	-8.50 [-15.39; -1.61]
Kolaitis et al, 2021	85.20	17.40	118	79.00	15.60	432	27.6	6.20 [2.73; 9.67]
Zamanian et al, 2018	80.80	13.20	90	81.40	14.10	97	26.9	-0.60 [-4.51; 3.31]
**Total (95% CI)**			**281**			**593**	**100.0**	**0.22 [-9.33; 9.78]**

Heterogeneity: $Tau^2 = 27.93$; $Chi^2 = 16.23$, df = 3 (P = 0.0010); $I^2 = 81.5\%$
Test for overall effect: $t_3 = 0.07$ (P = 0.9451)

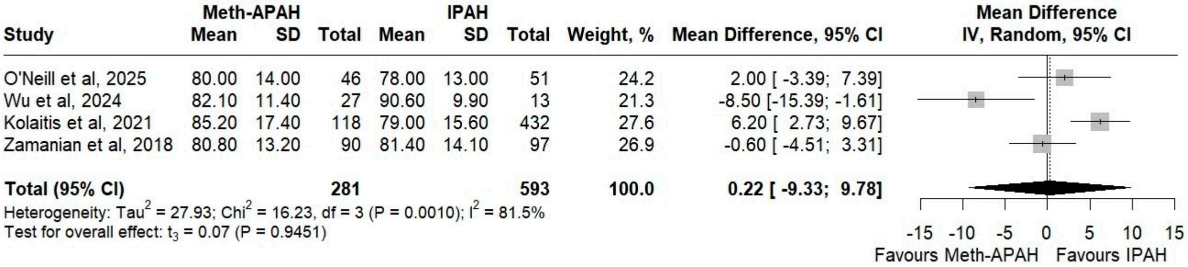

6-MWD: six-minute walk distance, 95% CI: 95% confidence interval, CI: cardiac index, HR: heart rate, IPAH: idiopathic pulmonary arterial hypertension, Meth-APAH: methamphetamine-associated pulmonary arterial hypertension, mPAP: mean pulmonary arterial pressure, PAWP: pulmonary artery wedge pressure, PVR: pulmonary vascular resistance, RAP: right atrial pressure, SD: standard deviation, SVI: stroke volume index, WHO-FC III/IV: World Health Organization functional classification 6
